# Gap junction protein connexin 36 proteostatic mechanisms in photoreceptor cells subjected to excitotoxic stress

**DOI:** 10.3389/abp.2026.16091

**Published:** 2026-08-14

**Authors:** Monika Katan, Klaudia Mróz, Anna Pacwa, Joanna Lewin-Kowalik, Adrian Smędowski

**Affiliations:** 1 The Laboratory for Translational Research in Ophthalmology, Department of Ophthalmology, Faculty of Medical Sciences in Katowice, Medical University of Silesia in Katowice, Katowice, Poland; 2 Department of Ophthalmology, Faculty of Medical Sciences in Katowice, Medical University of Silesia in Katowice, Katowice, Poland; 3 Department of Clinical Genetics and Rare Diseases, Faculty of Medical Sciences in Katowice, Medical University of Silesia in Katowice, Katowice, Poland; 4 GlaucoTech Co., Bytom, Poland; 5 Department of Ophthalmology, Professor K. Gibiński University Clinical Center, Faculty of Medical Sciences in Katowice, Medical University of Silesia in Katowice, Katowice, Poland; 6 Department of Pediatric Ophthalmology, Faculty of Medical Sciences in Katowice, Medical University of Silesia in Katowice, Katowice, Poland

**Keywords:** autophagy, central nervous system, connexins, excitotoxicity, gap junctions, retina, retinal degeneration

## Abstract

Connexin 36 (Cx36), a neuronal gap junction protein, is essential for retinal signal transmission but may also contribute to neurodegeneration by facilitating the spread of excitotoxic stress signals. This study aimed to examine the degradation pathway of Cx36 under stress conditions relevant to retinal disease. Using immortalized 661W murine photoreceptor cells transfected with GFP-tagged Cx36, we evaluated the effects of lysosomal inhibition (Bafilomycin A1) and excitotoxic stimulation (NMDA). Immunofluorescence revealed intracellular Cx36 accumulation following Bafilomycin and NMDA exposure, accompanied by increased expression of p62/SQSTM1 and PSMA1, suggesting impaired autophagic flux and a potential compensatory involvement of the proteasome. Elevated RAB11A levels suggested recruitment of endosomal recycling pathways. NMDA treatment was associated with increased ubiquitin accumulation, particularly in Cx36-overexpressing cells, indicating enhanced proteostatic stress under excitotoxic conditions. These findings highlight the stress-responsive behavior of Cx36 and suggest that connexin proteostasis may represent a potential target for future investigation in retinal neurodegeneration, including glaucoma and age-related macular degeneration. Immunofluorescence revealed intracellular accumulation of p62 and PSMA1 after Bafilomycin exposure with signal intensities increasing approximately 1.5-2-fold versus controls (n = 3, p < 0.05). These data are consistent with disrupted autophagic flux and suggest proteasome involvement, but do not constitute functional proof.

## Introduction

The retina is a direct extension of the central nervous system (CNS), derived from the embryonic diencephalon and retaining CNS-like architecture and physiology. Retinal ganglion cells (RGCs) are the retina’s sole output neurons, transmitting visual information via their axons, which form the optic nerve and synapses in thalamic and midbrain centers (Mayoral et al., 2018; Wong and Benowitz, 2022; Balraj et al., 2023; Soucy et al., 2023; Tsung et al., 2023). Like other CNS tracts, starting from the retrobulbar part, the optic nerve is myelinated by oligodendrocytes, features nodes of Ranvier and is enclosed in three meningeal layers.

In addition to their vertical output function, RGCs integrate inputs from retinal interneurons (horizontal and amacrine cells) and encode key visual features, including contrast, motion, and spatial patterns. Diverse subtypes of retinal neurons are essential for visual processing under normal and pathological conditions (Duan et al., 2015; Sanes and Masland, 2015; Ran et al., 2020). Due to their CNS nature, the retina and optic nerve lack regenerative capacity, and their dysfunction often results in irreversible vision loss (Wong and Benowitz, 2022; Tsung et al., 2023).

Signal conduction in RGC axons depends on voltage-gated ion channels at nodes of Ranvier and periaxonal ion regulation by glia. Following optic nerve injury, a resilient RGC subtype reduces excitability by downregulating sodium channels via a calcium-dependent mechanism, conserving energy and promoting survival (Werginz et al., 2014; Zapadka et al., 2025). The optic nerve transmits visual signals using both chemical and electrical modalities. Neurotransmitters like glutamate, GABA, and dopamine modulate RGC activity through ligand-gated channels and metabotropic receptors inducing different secondary messengers. It is now widely accepted that electrical coupling via gap junctions (GJs) formed by connexin (Cx) 36 and 45 further synchronizes neuronal firing and integrates visual signals (Söhl and Willecke, 2004; O’Brien and Bloomfield, 2018; González-Casanova et al., 2021; Maruyama, 2023).

GJs, including those formed by Cx36, enable electrical coupling between RGC axons, promoting synchronization and rapid conduction. These connections enhance temporal coding, and their disruption may modulate neurodegeneration processes (Akopian et al., 2017). Cx36 enables fast, synchronous transmission, critical for temporal precision, while Cx45 supports signal modulation and plasticity. Dysfunction of either affects visual processing and contributes to degeneration. Cx36 plays distinct roles in the retina: in the outer retina, photoreceptor gap junctions involving Cx36 mediate rod-cone coupling and regulate visual signal integration, whereas in the inner retina, Cx36 contributes to electrical coupling of retinal ganglion cells and amacrine cells. The present study, based on 661W photoreceptor-like cells, specifically addresses Cx36 biology in photoreceptor cells, without direct implications for whole retina coupling (Smedowski et al., 2020; Bloomfield and Völgyi, 2004; Salinas-Navarro et al., 2010; O’Brien and Bloomfield, 2018).

Connexin’s proteostasis, involving synthesis, trafficking, and degradation via lysosomal/autophagic and ubiquitin-proteasome pathways, is essential for proper GJs function. Connexins, like Cx36 and Cx43, have short half-lives and are regulated via phosphorylation and ubiquitination. Disrupted proteostasis in the retina is one of the essential mechanisms contributing to retinal neurodegenerative diseases development (Nielsen et al., 2012; Gong et al., 2016; Solan and Lampe, 2018; Yang et al., 2024). Ubiquitin regulates protein homeostasis in ocular tissues via the ubiquitin-proteasome system (UPS), influencing retinal development, stress responses, and pathomechanism of diseases such as glaucoma and age-related macular degeneration (Svikle et al., 2022; Ma et al., 2024; Wei et al., 2024).

By connecting retinal neurons of different functions, gap junctions are involved in retinal development, circadian rhythms, and neuroprotection. Their coupling strength is dynamically regulated through activity-dependent mechanisms, such as the modulation of AII amacrine cell networks by nonsynaptic NMDA receptor signaling, which enables adaptive changes in retinal processing. In addition, the expression of connexins that form these junctions is shaped by both intrinsic genetic programs and extrinsic environmental factors, emphasizing their central role in maintaining retinal homeostasis and function. GJs formed by connexins create cytoplasmic continuity between retinal neurons, enabling ion and small molecule metabolites exchange. In the retina, Cx36 and Cx45 structure electrical synapses between photoreceptors, amacrine cells, and RGCs. Both connexins contribute to signal transmission and exhibit significant plasticity, with Cx36 enhancing signal-to-noise ratio and firing synchrony. Importantly, Cx36 itself undergoes activity-dependent modulation through phosphorylation and other post-translational modifications, allowing adaptive changes in gap junctional coupling strength under physiological and pathological conditions (Wade Kothmann et al., 2012; Kovács-Öller et al., 2023; Söhl and Willecke, 2004; O’Brien and Bloomfield, 2018).

Excitotoxicity caused by N-methyl-D-aspartate (NMDA) receptor overactivation leads to calcium overload, oxidative stress, mitochondrial dysfunction, and apoptosis, contributing to neuronal damage in neurodegenerative conditions. Therapeutic interventions target NMDA receptors to prevent injury while preserving normal transmission (Aarts et al., 2002; Zhang et al., 2016; Binvignat and Olloquequi, 2020). Under excitotoxic conditions, Cx36 expression and membrane conductivity increases, facilitating the spread of calcium overload and pro-apoptotic signals through neuronal GJs. This contributes to accelerated neuronal death in ischemia and seizures, highlighting Cx36 as a potential therapeutic target (Frantseva et al., 2002; Belousov et al., 2017).

There is no immortalized neuronal cell line available as a model for studying neurodegeneration processes. The immortalized 661W cells represent murine cone photoreceptor-derived cells expressing cone-specific markers (e.g., cone opsins, cone arrestin) and lacking rod features. It shows neuronal morphology without forming outer segments, limiting studies on light-driven trafficking. Nevertheless, 661W cells retain light responsiveness through cGMP modulation and activation of canonical phototransduction pathways, making them useful for investigating cone degeneration and phototransduction (Natoli et al., 2016; Lin et al., 2020; Zhang et al., 2023). Transcriptomic profiling has confirmed the expression of cilia-associated genes and the presence of functional primary cilia, validating 661W as a model for studying ciliopathies. Compared to other neuron-like cell lines, e.g., neuroblastoma cell lines, 661W cells provide a more physiologically relevant retinal phenotype and greater light responsiveness, making them preferable for modeling retinal diseases affecting photoreceptor cells (Wheway et al., 2019; Brunet et al., 2025). The murine 661W photoreceptor cell line provides a useful *in vitro* model for studying connexin biology in photoreceptor-like cells, although findings should be interpreted cautiously due to differences from intact retinal tissue (González-Casanova et al., 2021).

Photoreceptor connexins, predominantly Cx36 in mammalian rods and cones, mediate rod-cone coupling and contribute to visual signal integration in the outer retina (Deans et al., 2002; Wheway et al., 2019; Kovács-Öller et al., 2017). Cx36-containing gap junctions in photoreceptors are dynamically regulated by phosphorylation and neuromodulators (e.g., dopamine, adenosine), as well as by circadian mechanisms, enabling light- and state-dependent control of electrical coupling, which in turn adjusts the sensitivity and signal-to-noise properties of photoreceptor outputs (Zhang et al., 2015; Zhang S. et al., 2020).

In this study, we aimed to investigate the role of Cx36 in proteostatic regulation under stress conditions, with particular focus on its involvement in autophagy and protein degradation pathways in photoreceptor cells. Our project explores the concept of modulating connexin degradation to study retinal cell survival. This research may provide new insights into the molecular mechanisms underlying the development and progression of retinal neurodegenerative diseases (Salinas-Navarro et al., 2010; Ma et al., 2024).

**FIGURE 1:**
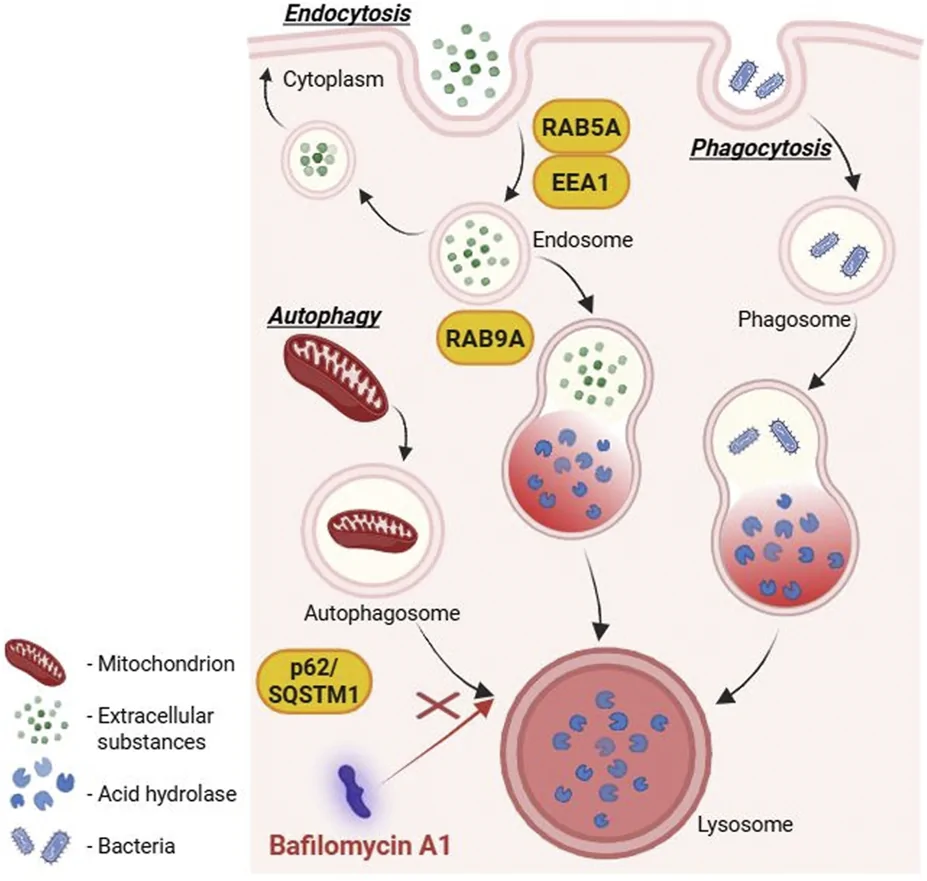
Diagram of the action mechanism of the autophagy-lysosome pathway. Figure was created by using BioRender.com.

**FIGURE 2:**
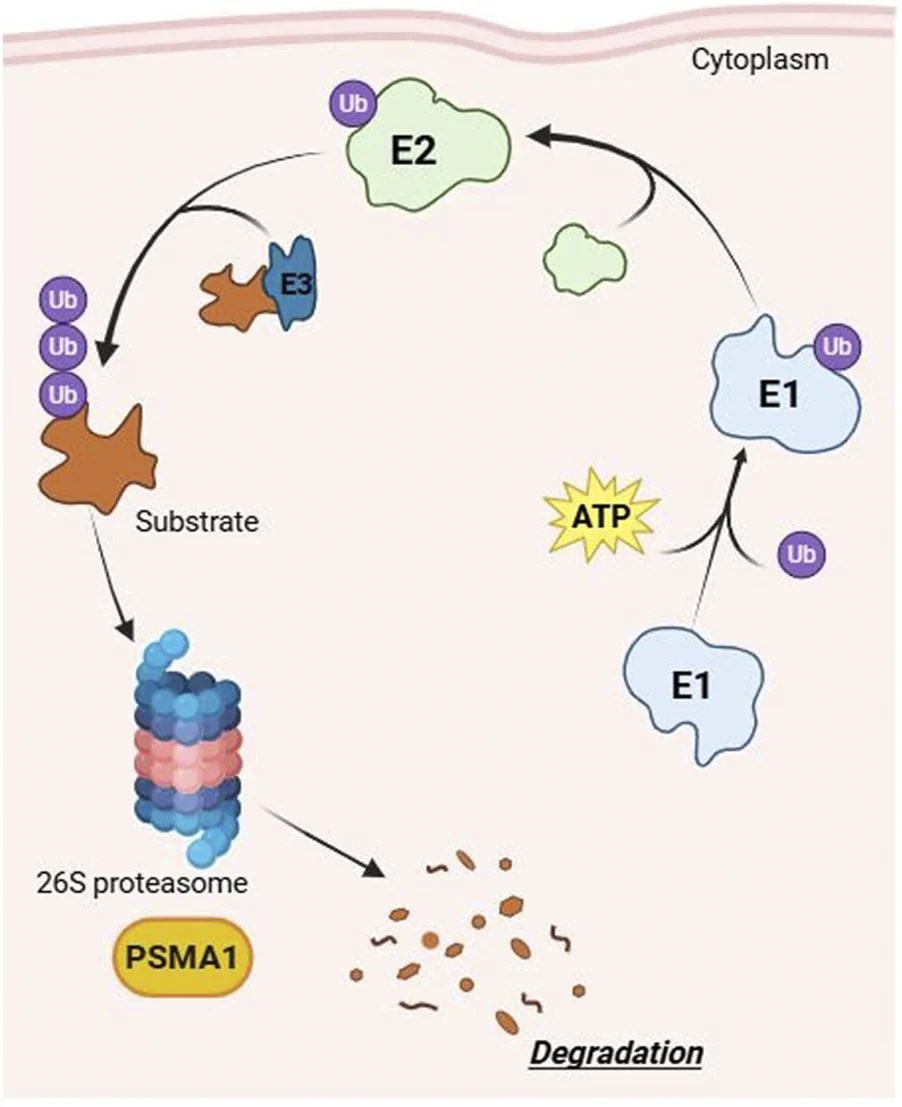
Diagram of the action mechanism of UPS. Figure was created by using BioRender.com.

Eukaryotic cells maintain proteostasis through two primary proteolytic systems: the autophagy-lysosome pathway (Figure 1) and the ubiquitin-proteasome system (UPS) (Figure 2). The UPS is responsible for the selective degradation of short-lived or misfolded proteins via polyubiquitination and subsequent recognition by the proteasome, playing a particularly critical role under cellular stress conditions. In parallel, autophagy facilitates the degradation of long-lived proteins, protein aggregates, and dysfunctional organelles by sequestering them into autophagosomes, which then fuse with lysosomes for enzymatic breakdown. RAB11A controls the return of internalized receptors from recycling endosomes to the plasma membrane and contributes to the regulation of vesicular trafficking processes underlying endosomal sorting. Lysosomes facilitate the degradation of a wide range of biological substrates through endocytosis, phagocytosis, and autophagy. This includes extracellular proteins, large extracellular entities such as pathogens and apoptotic cells, as well as intracellular components such as misfolded or aggregated proteins, dysfunctional organelles, and intracellular pathogens. p62/SQSTM1 serves as a selective autophagy receptor by binding polyubiquitinated protein aggregates in the cytoplasm and delivers them to the autophagosome via interaction with LC3, facilitating subsequent degradation upon fusion with the lysosome. EEA1 is an early endosome-associated protein that plays a critical role in tethering and fusion of vesicles to early endosomes, acting downstream of RAB5A, which regulates early endocytic trafficking and endosome biogenesis. RAB5A is a small GTPase essential for early endosome formation and cargo internalization. RAB9A regulates retrograde trafficking from late endosomes to the trans-Golgi network, contributing to the recycling of mannose-6-phosphate receptors and lysosomal enzyme sorting. ATP facilitates the activation of ubiquitin by the E1 ubiquitin-activating enzyme through covalent attachment. The activated ubiquitin is then transferred to an E2 ubiquitin-conjugating enzyme. The E3 ubiquitin ligase then mediates the transfer of ubiquitin to the target substrate by interacting with the E2 enzyme. The polyubiquitinated substrate is ultimately recognized and degraded by the 26S proteasome. PSMA1 is a structural subunit of the 20S core particle within the 26S proteasome complex, responsible for the ATP-dependent degradation of polyubiquitinated substrates. It contributes to the gating mechanism that regulates substrate entry (Sun-Wang et al., 2020; Li et al., 2022; Feng et al., 2024). Bafilomycin A1, a macrolide antibiotic, disrupts autophagic flux by interfering with two key stages of the autophagy-lysosome pathway.

First, it blocks V-ATPase-dependent acidification of lysosomes, thereby impairing the activity of lysosomal hydrolases. Second, it inhibits autophagosome-lysosome fusion, potentially by affecting Ca-P60A/SERCA-mediated calcium transport (Klionsky et al., 2008; Mauvezin and Neufeld, 2015). As a result, autophagosomes accumulate within the cytoplasm, reflecting an arrest of the autophagic degradation process.

Eeyarestatin I (ESI) is a small-molecule inhibitor that selectively disrupts the endoplasmic reticulum-associated degradation (ERAD) pathway, thereby impairing the clearance of misfolded proteins within the endoplasmic reticulum (ER). ERAD is a fundamental protein quality control system that identifies, ubiquitinates, and retrotranslocates abnormal ER-resident proteins to the cytosol, where they are targeted for degradation by the proteasome-thereby maintaining ER proteostasis. Inhibition of ERAD by ESI leads to the accumulation of misfolded proteins, triggering ER stress signaling pathways and demonstrating anti-proliferative effects in colorectal cancer models (Ruggiano et al., 2014; Krshnan et al., 2022; Peng et al., 2025).

The aim of this study is to investigate the mechanisms regulating the proteostasis of Cx36 in retinal photoreceptor cells exposed to excitotoxic stress. To address this objective, we first employed photoreceptor cells with increased expression of Cx36 to facilitate the analysis of its turnover. Subsequently, the lysosomal pathway was inhibited using bafilomycin in order to assess its contribution to Cx36 degradation. Finally, immunofluorescent staining of specific molecular markers was performed to evaluate the involvement of distinct proteostatic pathways in regulating Cx36 levels.

## Materials and methods

### *In vitro* study design

Connexin 36, a neuronal gap junction protein, undergoes intracellular degradation via both the proteasomal and endosomal pathways. Inhibition of these degradation pathways results in the intracellular accumulation and aggregation of connexins, which may affect cell viability. In this study, we utilized immortalized 661W mouse photoreceptor cells. Once cells reached appropriate confluency, transfection was performed using plasmids encoding GFP-tagged connexin 36, followed by pharmacological inhibition of the lysosomal pathways using Bafilomycin A1. Immunofluorescence was used to assess the expression of key markers associated with autophagy (p62), endocytosis (EEA1, RAB5A, RAB9A, RAB11A), and proteasomal degradation (PSMA1), in order to better understand the mechanisms involved in intracellular connexin trafficking. To evaluate connexin aggregation under neurodegenerative stress, cells were additionally exposed to NMDA to induce excitotoxicity. Ubiquitin, a specific marker of proteolysis and protein aggregation, was used to evaluate degradation process, while Cx36 staining was performed to assess its expression pattern.

#### Cell culture

Mouse immortalized photoreceptor 661W cells, kindly donated by Dr. Muayyad R. Al-Ubaidi (University of Houston, Houston, TX, USA), were stained with anti-arrestin antibody (Santa Cruz, 1:300) as a marker for photoreceptor cells (data not shown). The cells were seeded at a density of 5,000 cells per well on round glass coverslips in 24-well plates containing high-glucose Advanced DMEM (Gibco), supplemented with 10% fetal bovine serum (FBS; PanBiotech) and 1% penicillin-streptomycin (P/S; Gibco). Cultures were maintained at 37 °C in a humidified 5% CO_2_ atmosphere, and the medium was replaced every 3 days. Cell confluence was monitored daily by phase-contrast microscopy, and transfection was performed when cells reached approximately 50% confluence.

#### Transfection with plasmids

An in-house generated plasmid encoding Cx36-GFP under the control of a constitutive promoter was used for transfection to visualize intracellular Cx36 distribution in 661W cells. Plasmids are commonly used for gene expression and can be assembled using homology-based methods such as Gibson assembly (Azizi et al., 2015; Nora et al., 2019; Blanch-Asensio et al., 2023).

Cells were plated on glass coverslips in 24-well plates as described above. For transfection, the fully supplemented medium was replaced with serum-reduced, antibiotic-free Advanced DMEM containing 5% FBS. Transfection was performed using Lipofectamine™ 3000 (Thermo Fisher Scientific) according to the manufacturer’s instructions. Plasmids encoding GFP-tagged connexins (Cx36-GFP) were used at a concentration of 100 ng/μL, with 500 ng (5 μL) of plasmid DNA added per well. The Master mix of DNA solution was prepared by mixing plasmid DNA Cx36-GFP with transfection medium and Lipofectamine 3000 Transfection Reagent (P3000 Reagent). The mixture was incubated for 20 min at room temperature to allow complex formation, then added to the cells. Control wells received transfection reagent without DNA. Cells were incubated with the transfection medium for 24 h at 37 °C and 5% CO_2_. Transfection efficiency was evaluated under a fluorescence microscope, after which the medium was replaced with fresh, fully supplemented medium and cells were cultured for next 24 h. This approach enabled visualization of intracellular distribution and accumulation patterns of Cx36 under proteostatic stress. Cx36-GFP overexpression was employed to facilitate robust visualization of connexin subcellular localization and aggregation within photoreceptor cells. As a control, non-transfected cells were included in each experimental set to assess baseline expression and localization of endogenous proteins.

#### Lysosomal pathways blockage with bafilomycin

To inhibit autophagic flux, 24 h after transfection, the medium was replaced with a fresh, fully supplemented medium containing 100 nM Bafilomycin A1 (Sigma, St. Louis, MO, USA). Untreated transfected cells were used as controls. Control cells for the bafilomycin experiments were processed in parallel and fixed at the same experimental endpoint as treated cells. Cells were incubated for 12 h, then fixed with ice-cold 5% paraformaldehyde and prepared for downstream immunofluorescence staining to assess autophagic, lysosomal and endosomal pathway modulation.

#### Excitotoxicity induction with NMDA

Excitotoxic stress was induced by exposing cells to NMDA (Sigma, St. Louis, MO, USA). 24 h after transfection, medium was exchanged for fresh fully supplemented medium containing 100 μM NMDA and cells were incubated for 4 h under standard conditions (37 °C, 5% CO_2_). NMDA was freshly diluted prior to use to ensure stability and efficacy. Untreated transfected cells served as negative controls. Control cells for the NMDA experiments were processed in parallel and collected at the same experimental endpoint as NMDA-treated cells.

#### Immunostaining

For immunofluorescence, cells were rinsed with sterile 0.1 M PBS and fixed in 4% paraformaldehyde (PFA) for 1 h at 4 °C. Cells were then washed twice in TBS to remove fixative residues and incubated with a blocking solution containing 10% NGS in TBST buffer (TBS +0.1% Triton X) for 30 min.

Transfected cells treated with NMDA were stained with primary antibodies against Cx36 (Santa Cruz, 1:100) and ubiquitin (Santa Cruz, 1:100). Bafilomycin-treated cells were stained with primary antibodies as follows: SQSTM1/p62 (Santa Cruz, 1:300), P4D1 (Santa Cruz, 1:300), PSMA1 (Invitrogen, 1:300), EEA1 (Santa Cruz, 1:300), RAB5A, RAB9A, and RAB11A (Santa Cruz, 1:300). Primary antibodies were diluted in 1% NGS/TBST and incubated for 48 h at 4 °C. After primary incubation, cells were washed three times in TBS and incubated for 3 h at room temperature with Alexa Fluor 594-conjugated secondary antibody (1:500, Life Technologies, Carlsbad, CA, USA). Cells were washed and mounted using Mowiol containing DAPI for nuclear counterstaining. The images were taken using fluorescent microscopy AxioScope (Zeiss, Germany).

After primary antibody incubation, cells were washed three times with TBS and incubated for 3 h at room temperature with Alexa Fluor 594-conjugated secondary antibody (1:500, Life Technologies, Carlsbad, CA, USA). Cells were washed and mounted with Mowiol containing DAPI for nuclear counterstaining. Fluorescent images were acquired using an AxioScope microscope (Zeiss, Germany) with a ×40 objective under identical acquisition settings for all experimental groups, including magnification, exposure time, and detector parameters. Quantification of the immunofluorescence signal was performed on microscopic images using ImageJ software. Mean fluorescence intensity was measured within manually defined regions of interest (ROIs), each corresponding to a single cell, with background subtraction performed prior to analysis. For each experimental group, three independent biological replicates were conducted, with approximately 5 ROIs corresponding to individual cells analyzed per replicate. Independent biological replicates were used as the statistical unit. Image acquisition and fluorescence quantification were performed using identical settings for all groups; and the analysis was performed in a blinded manner. Transfected cells were identified as double-positive for Cx36 and a specific marker protein (Rab11a, PSMA, p62, EEA1, Rab5a, or Rab9a), while non-transfected cells exhibited marker-specific staining without Cx36 expression. Statistical comparisons were performed using one-way ANOVA followed by Šidák’s *post hoc* test on data derived from the independent biological replicates.

## Results

### Transfection efficacy

**FIGURE 3:**
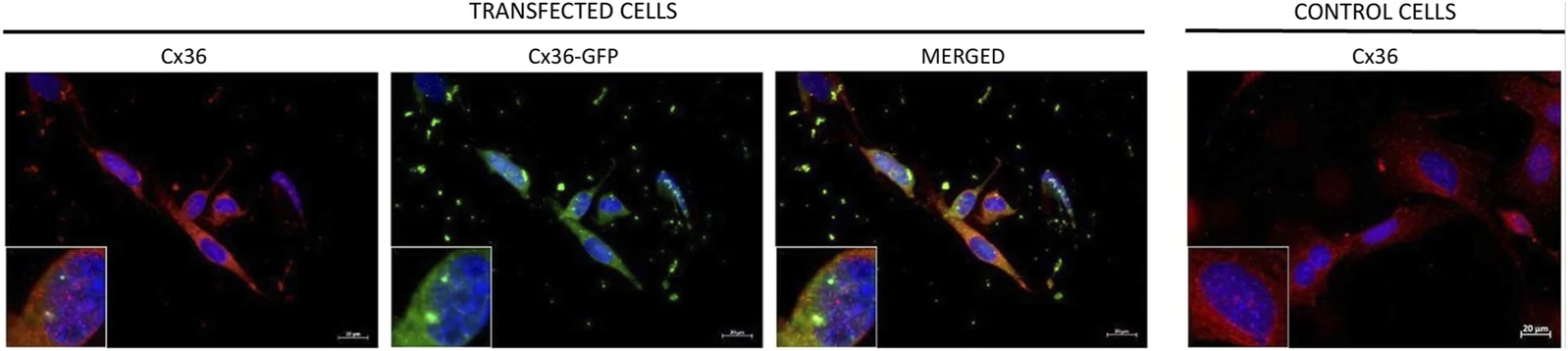
Transfection of 661W cells. Red - Cx36, green - Cx36-GFP, blue - DAPI.

**FIGURE 4:**
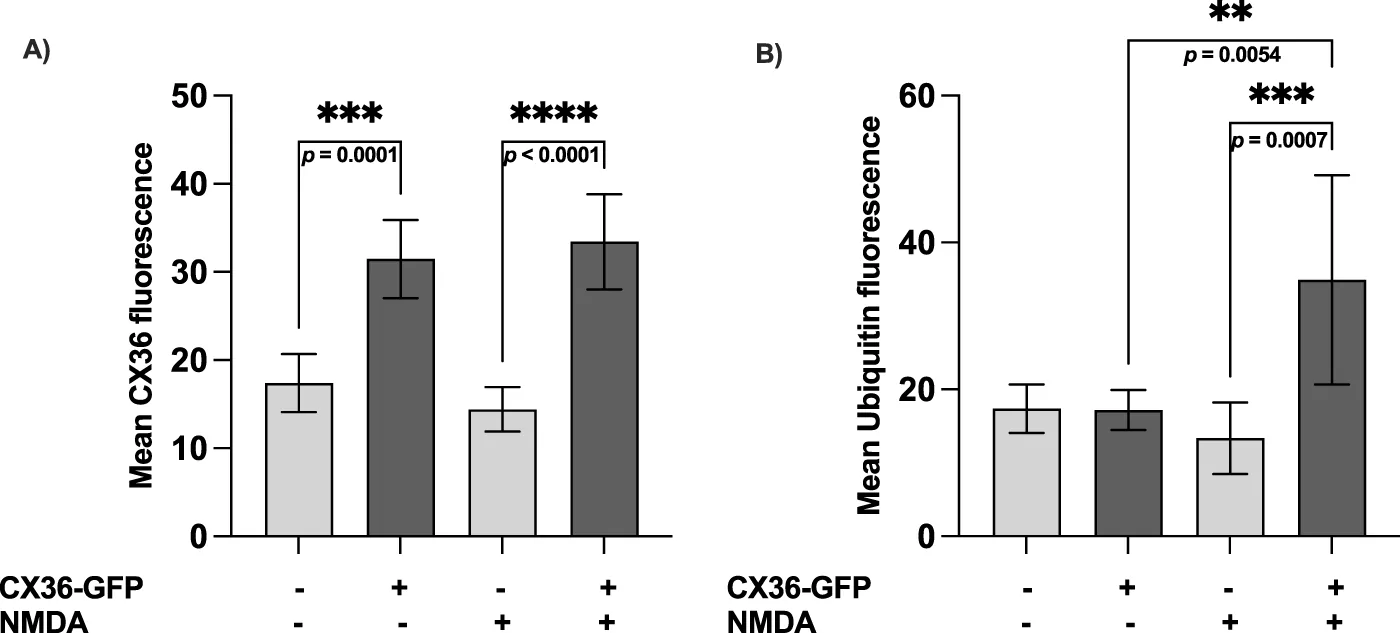
Quantification of immunofluorescence signal intensity for Cx36 and ubiquitin in 661W cells exposed to NMDA. **(A)** Mean Cx36 fluorescence intensity. **(B)** Mean ubiquitin fluorescence intensity. Mean fluorescence intensity was measured in 661W cells under four conditions: control, Cx36-GFP transfection, NMDA treatment, and combined Cx36-GFP transfection with NMDA treatment. Cx36-GFP: (+), transfected cells; (–), control cells; NMDA: (+), treated cells; (–), untreated cells. Mean values were calculated from ROIs, with each ROI corresponding to a single cell. Data are presented as mean ± SD (n = 6). Statistical analysis was performed using one-way ANOVA, with p < 0.05 considered significant. The exact p-values are shown on the graphs.

661W cells exposed to plasmids encoding Cx36 tagged with GFP exhibited an intense green fluorescent signal, whereas no fluorescence was detected in the control group (Figure 3). The presence of GFP fluorescence in transfected cells confirms the effectiveness of the transfection procedure and validates transcription of the introduced plasmid DNA, suggesting efficient delivery and expression of the Cx36-GFP fusion protein in 661W cells. Immunostaining confirmed elevated levels of Cx36 expression in transfected cells compared to controls (Figure 3). Transfection efficiency was approximately 15%, as determined by the proportion of GFP-positive cells among the total cell population (n = 3 independent biological replicates). Quantitative analysis of Cx36 fluorescence intensity in transfected versus non-transfected cells (without NMDA treatment) is presented in Figure 4A. Control, non-transfected cells exhibited only low baseline Cx36 signal, confirming that the detected fluorescence in transfected samples primarily reflects overexpressed Cx36-GFP.

### Bafilomycin-induced expression of degradation markers

**FIGURE 5:**
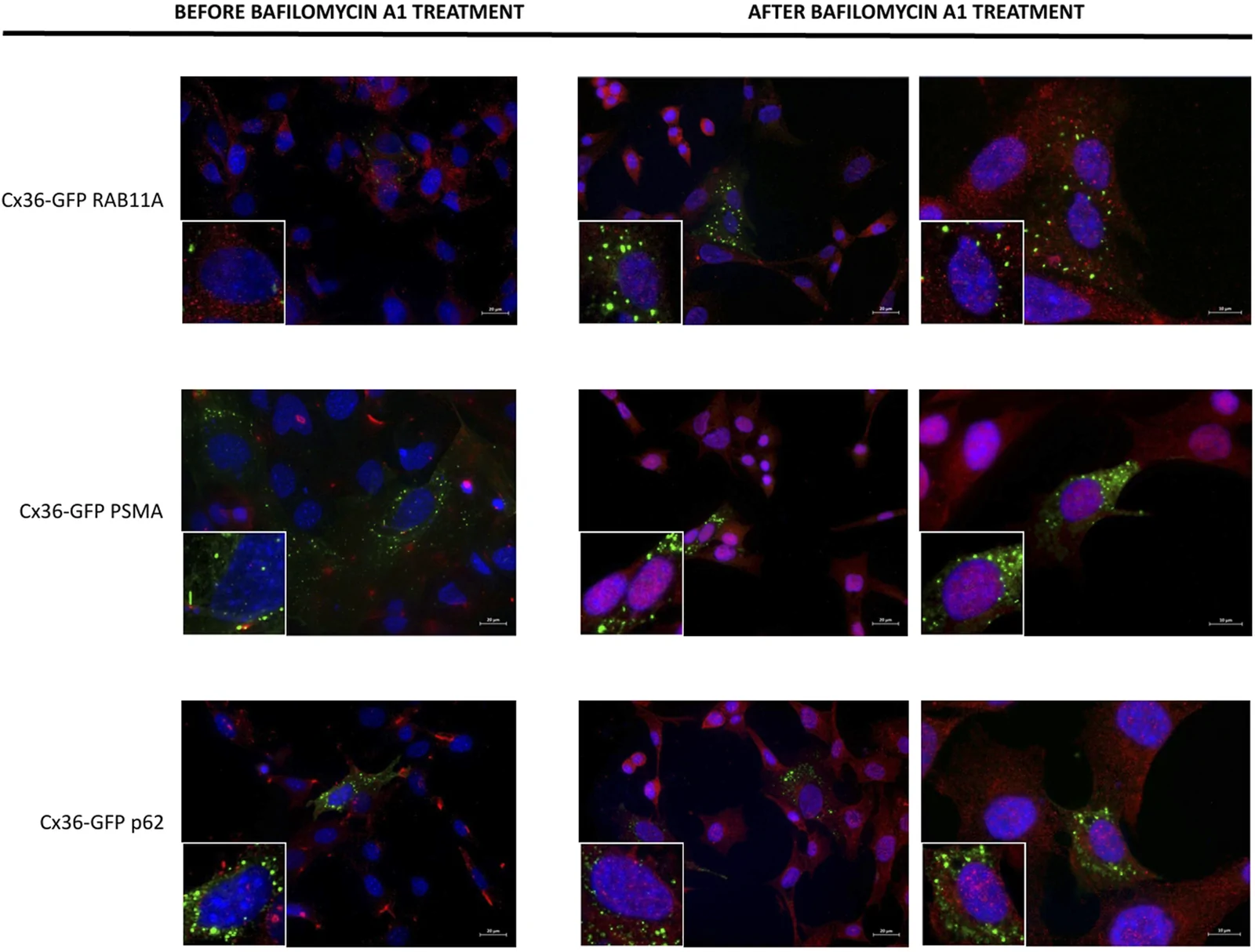
Bafilomycin-induced expression of degradation markers. Green - Cx36-GFP, red - specific marker (RAB11A, PSMA1, p62), blue - DAPI.

**FIGURE 6:**
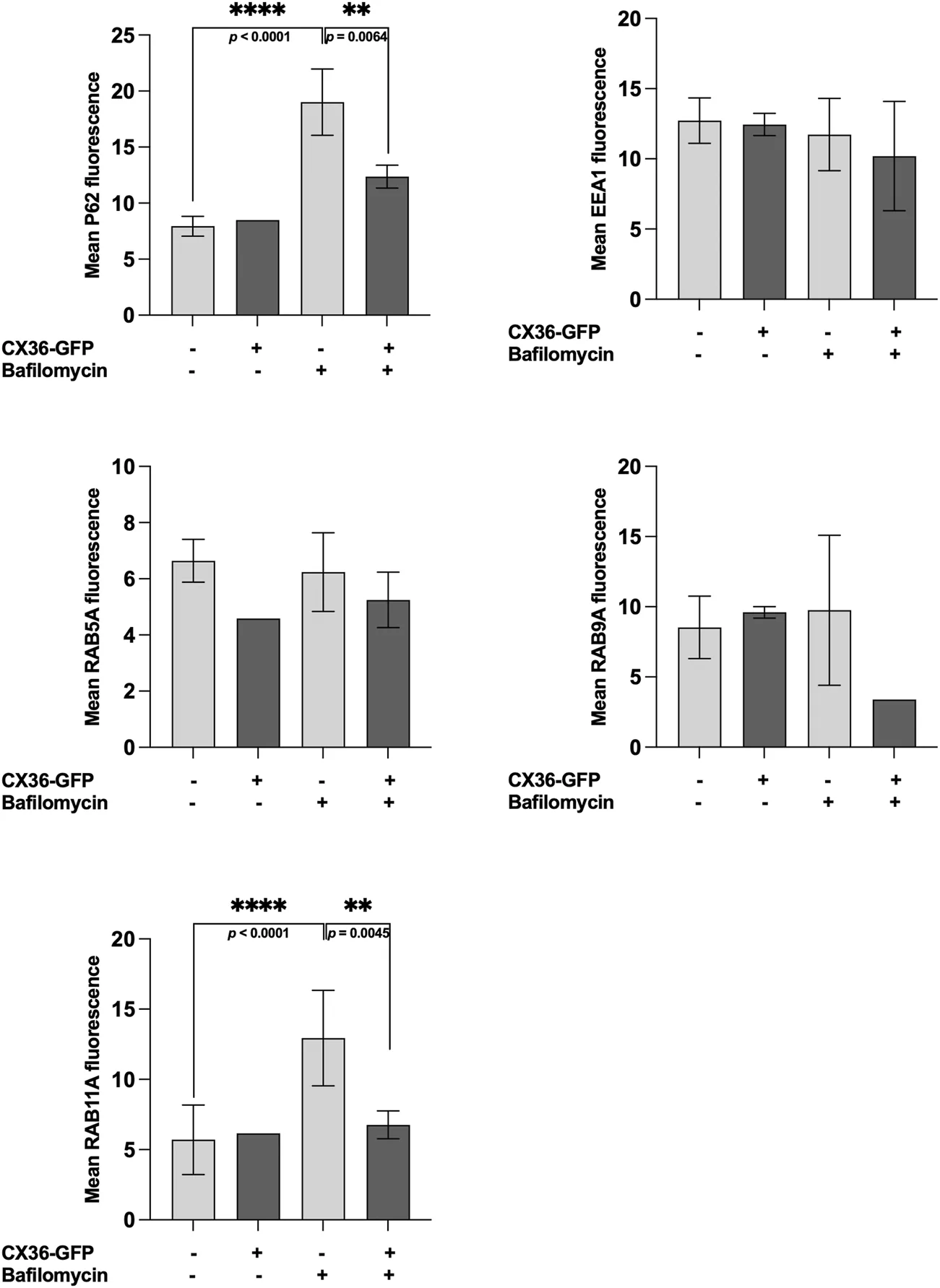
Quantification of immunofluorescence signal intensity for selected proteins involved in protein degradation pathways. Mean fluorescence intensity of p62, EEA1, RAB5A, RAB9A, and RAB11A was measured in 661W cells under four conditions: control, Cx36-GFP transfection, bafilomycin treatment, and combined Cx36-GFP transfection with bafilomycin treatment. (Cx36-GFP: (+) transfected cells, (−) control cells; bafilomycin: (+) treated cells, (−) untreated cells) The mean values were calculated from ROIs, with each ROI corresponding to a single cell. Data are presented as mean ± SD; *n* = 3, statistical analysis was performed using one-way ANOVA, with *p* < 0.05 considered significant. The exact *p*-values are shown on the graphs.

**FIGURE 7:**
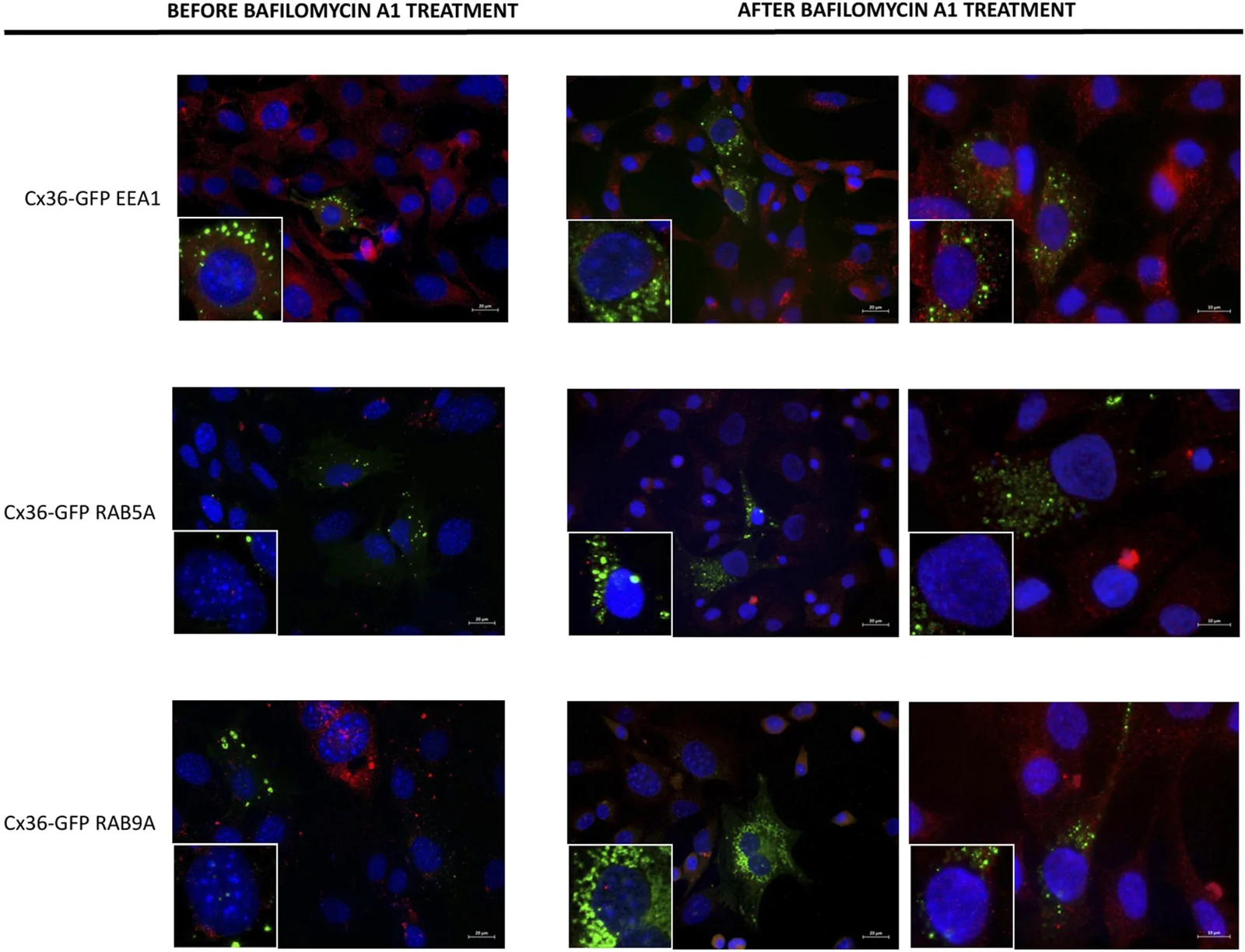
Bafilomycin-induced expression of degradation markers. Green - Cx36-GFP, red - specific marker (EEA1, RAB5A, RAB9A), blue - DAPI.

661W cells were transfected and subsequently treated with Bafilomycin A1 to investigate the expression of key markers involved in intracellular degradation pathways. Immunostaining revealed increased levels of the autophagosome adaptor protein SQSTM1/p62 relative to controls (Figure 5, lower panel and Figure 6). Interestingly, although p62 levels increased following bafilomycin treatment, a relative decrease in p62 signal intensity was observed in Cx36-transfected cells treated with bafilomycin compared to cells treated with bafilomycin alone. Increased p62 expression after bafilomycin treatment indicates accumulation of autophagic cargo due to impaired autophagic flux, as p62 is normally degraded during functional autophagy. Although definitive confirmation would require LC3-II turnover analysis, which was not performed in this study (Figure 5, lower panel and Figure 6). The observed increase in PSMA1 expresses a potential compensatory involvement of the proteasome, although functional activation was not directly assessed; however, without functional activity assays, this should be considered indicative rather than definitive evidence of UPS potential involvement (Figure 5, middle panel). Moreover, RAB11A, a small GTPase associated with the recycling endosome pathway and endocytic trafficking, showed increased expression, which may reflect altered regulation of endosomal recycling pathways in response to impaired protein degradation (Figure 5, upper panel and Figure 6). RAB11A expression was increased, which may reflect enhanced endosomal recycling activity in response to impaired protein degradation. Additionally, we did not observe any changes in the accumulation of proteins such as EEA1, RAB5A, or RAB9A following treatment with bafilomycin, nor after transfection with Cx36-GFP (Figures 6, 7). Data are presented as mean ± SD; n = 3 independent biological replicates.

### NMDA insult

Transfected cells were subjected to immunostaining for Cx36 and ubiquitin to evaluate transfection efficiency and assess the intracellular stability of the expressed connexin 36. Transfected cells exhibited a statistically higher expression of Cx36, suggesting efficient transfection (Figure 4A).

**FIGURE 8:**
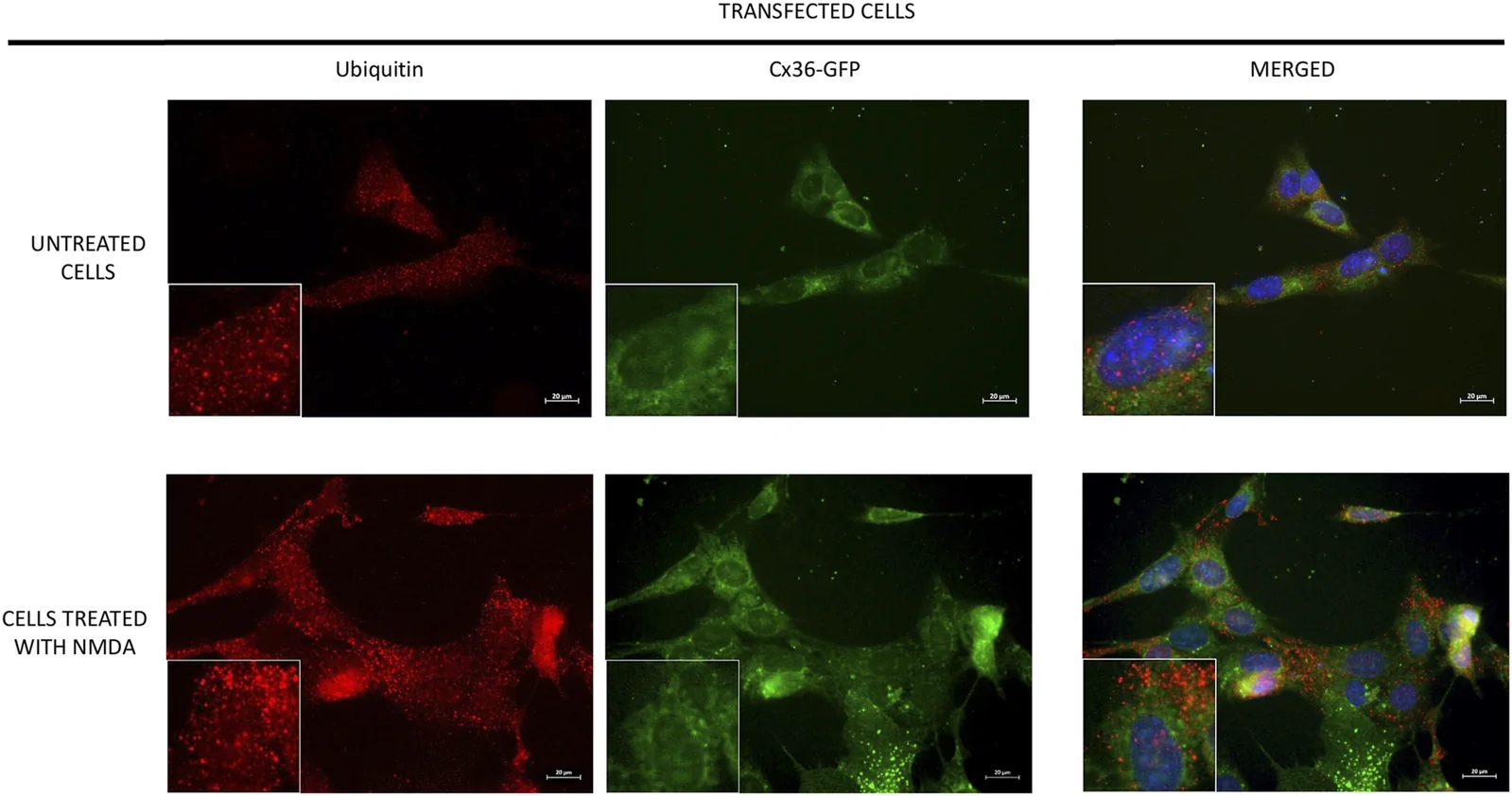
The effect of NMDA treatment on 661W cells after Cx36-plasmid transfection. Transfected cells and transfected cells treated with NMDA (red - ubiquitin, green - Cx36-GFP, blue - DAPI).

**FIGURE 9:**
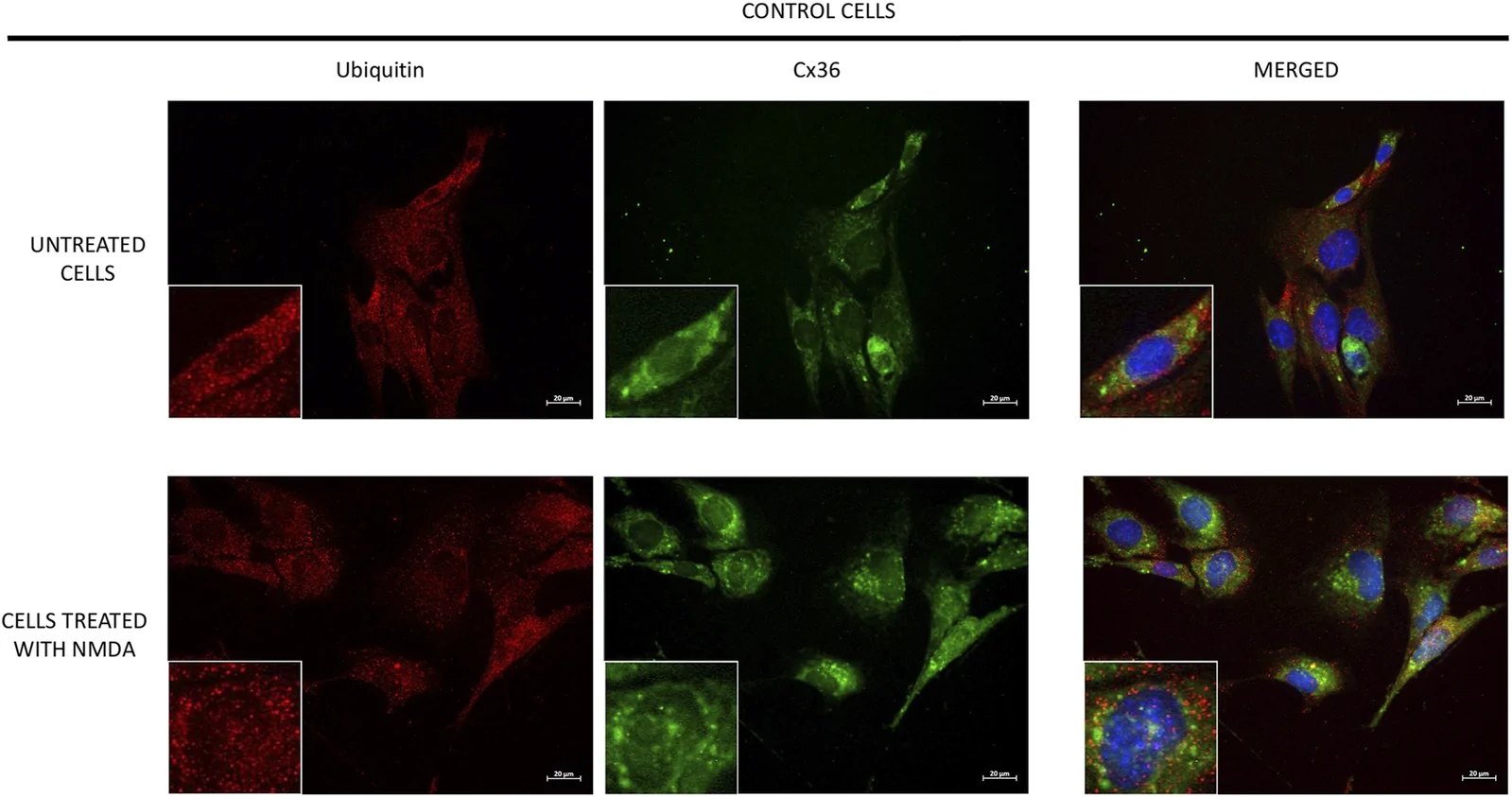
The effect of NMDA treatment on 661W cells after Cx36-plasmid transfection. Control cells without transfection and control cells without transfection treated with NMDA (red - ubiquitin, green - Cx36, blue - DAPI).

Ubiquitin staining (Figure 8) showed the presence of ubiquitin aggregates, with signal intensity comparable to that observed in non-transfected cells (Figure 9), suggesting no significant increase in proteostatic stress under basal conditions (Figure 4B).

**FIGURE 10:**
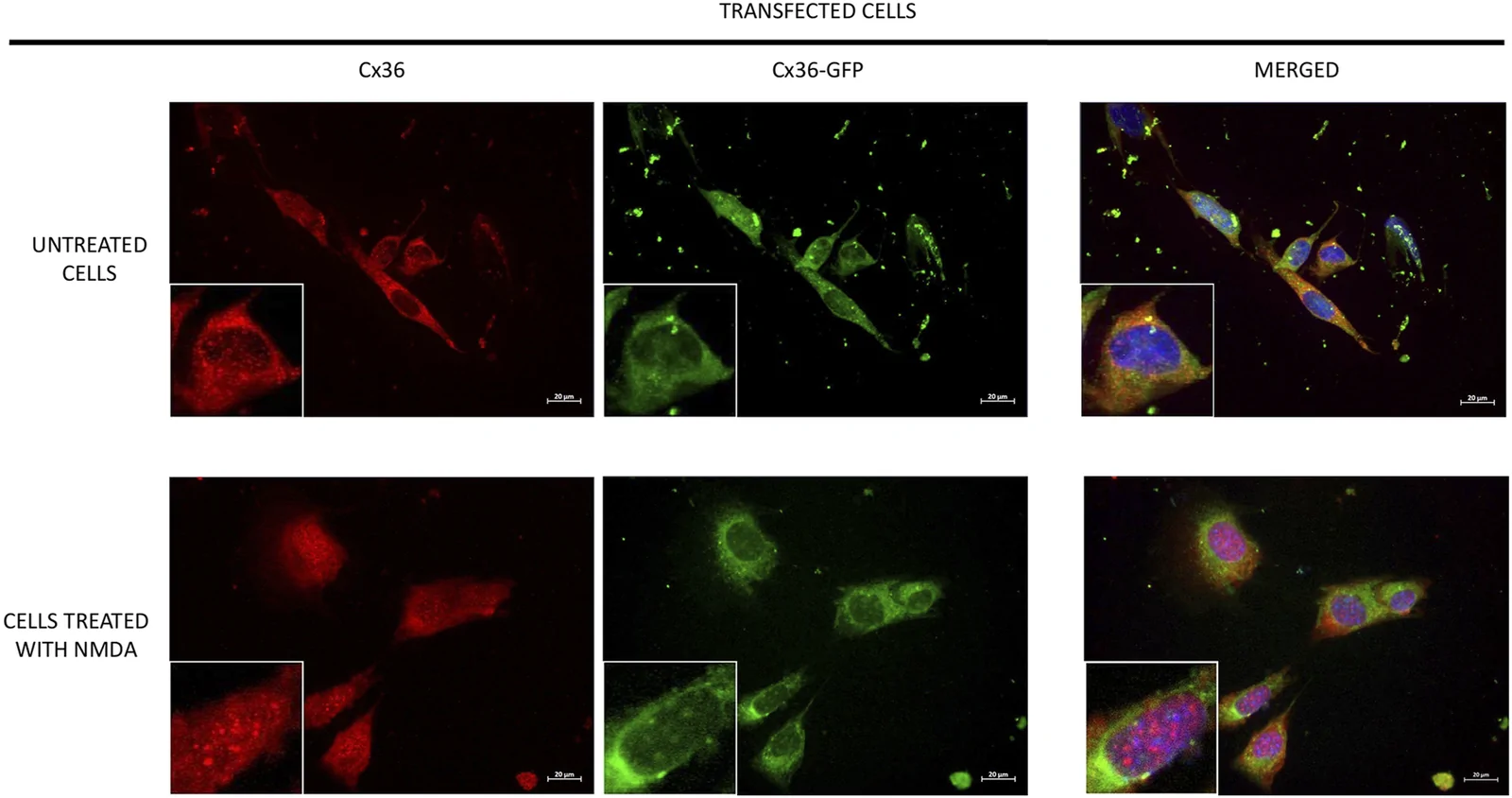
The effect of NMDA treatment on 661W cells after Cx36-plasmid transfection. Transfected cells and transfected cells treated with NMDA (red - Cx36, green - Cx36-GFP, blue - DAPI).

Analogous staining was performed on cells incubated with NMDA. Representative images of Cx36 and Cx36-GFP distribution in transfected cells, with and without NMDA treatment, are shown in Figure 10. The Cx36 signal was comparable to that observed in transfected cells without NMDA treatment; however, a marked increase in ubiquitin staining intensity was detected in transfected cells exposed to NMDA compared to transfected cells alone. NMDA treatment also led to higher ubiquitin levels in transfected cells than in non-transfected controls, suggesting that NMDA exposure may enhance proteostatic stress particularly in Cx36-expressing cells (Figure 4B). Cx36 immunoreactivity in transfected cells was predominantly observed in the cytoplasm, with a punctate distribution pattern suggestive of intracellular vesicular localization. No major redistribution of Cx36 signal was observed following NMDA treatment.

## Discussion

Under excitotoxic stress, Cx36 expression in neuronal cells did not show a marked increase; however, NMDA treatment led to enhanced ubiquitin accumulation, particularly in Cx36-transfected cells. These findings suggest that excitotoxic conditions compromise Cx36 stability and promote its degradation. Increased ubiquitin levels further indicate activation of cellular mechanisms responsible for the removal of damaged or misfolded proteins. Therefore, modulation of Cx36 turnover or stabilization, rather than its simple upregulation, may represent a more effective strategy for mitigating excitotoxic injury and developing neuroprotective interventions (Ivanova et al., 2015). This notion aligns with previous observations that modulation of Cx36 turnover, rather than its absolute expression, plays a pivotal role in neuronal resilience. It is important to note that Cx36 aggregation has not been reported in any known neurodegenerative condition. In this study, Cx36 overexpression was used as an experimental strategy to overcome its short physiological half-life and to facilitate analysis of intracellular handling under defined stress conditions. This cytoplasmic distribution likely reflects overexpression-driven intracellular accumulation and retention within the endoplasmic reticulum or vesicular compartments, rather than functional gap junction localization at the plasma membrane. This model does not reflect a pathological aggregation process *in vivo* but provides a controlled context for studying connexin proteostasis and the potential for cytoplasmic retention under stress. Notably, while functional connexins are characterized by a very rapid turnover, misfolded or non-junctional connexin species can persist much longer within the cell, forming cytoplasmic accumulations derived from proteins retained in the ER. Similar phenomena have been described for other connexins, such as the cataract-associated mutant Cx50P88S, which forms long-lived cytoplasmic aggregates following ER retention (Lichtenstein et al., 2008).

Blocking GJs under excitotoxic or ischemic conditions has been shown to significantly improve the survival of RGCs, with studies reporting up to a 70% increase in cell viability (Akopian et al., 2014). This notable protective effect underscores the key role of intercellular communication in the propagation of neuronal injury. Gap junctions, formed by connexin proteins, enable the direct exchange of ions and small molecules between adjacent cells, a process that under pathological conditions can facilitate the spread of toxic signals from injured to healthy neurons (Andrade-Rozental et al., 2000). By pharmacological inhibition of these channels or genetic silencing of specific connexins, it should be possible to interrupt this detrimental signaling cascade.

Genetic knockout studies of connexins Cx36 have provided valuable insights into their distinct contributions to neurodegeneration. Elimination of Cx36, which is predominantly expressed in neurons, has been found to significantly reduce RGC death under excitotoxic stress, where excessive glutamate release leads to calcium overload and oxidative damage. These findings suggest that individual connexins may contribute to neuronal vulnerability in a context-dependent manner, with each playing a unique role depending on the type of insult (Akopian et al., 2014). Immunohistochemical analyses further support this differential involvement, revealing that Cx36 exhibits distinct expression profiles and intracellular localization patterns under pathological stress conditions. In our model, NMDA-induced excitotoxic stress did not lead to a clear increase in Cx36 expression. Instead, the observed effects were associated with enhanced proteostatic stress responses, particularly in Cx36-overexpressing cells. No colocalization analyses with autophagic or proteostatic markers were performed, which limits the ability to directly link Cx36 distribution with specific intracellular compartments.

Immunostaining for the autophagy adaptor protein SQSTM1/p62 showed elevated expression levels compared to controls. This is consistent with impaired autophagic flux, although definitive confirmation would require LC3-II turnover analysis, which was not performed in this study. Under normal autophagy, p62 is selectively sequestered into autophagosomes and degraded upon fusion with lysosomes. When autophagic flux is impaired-due to lysosomal dysfunction or failed autophagosome-lysosome fusion-p62 degradation is blocked, resulting in its intracellular accumulation. This phenomenon has been documented across various contexts, including lipid-induced endoplasmic reticulum stress and nanoparticle exposure, where increased p62 staining correlates with autophagic blockade and cellular pathology (González-Rodríguez et al., 2014; Wu et al., 2020).

Lysosomes represent the terminal destination in the autophagic pathway, facilitating degradation of sequestered cargo through fusion with autophagosomes. Inhibition of this terminal stage by bafilomycin A1-an inhibitor of vacuolar H^+^-ATPase-prevents acidification and blocks autophagosome-lysosome fusion, thereby halting autophagic flux. The adaptor protein SQSTM1/p62 binds polyubiquitinated proteins and facilitates their delivery to autophagosomes, after which p62 itself is degraded within lysosomes under basal conditions. However, bafilomycin A1 treatment disrupts lysosomal degradation, resulting in a striking intracellular accumulation of p62 due to impaired clearance. This pharmacologically induced blockade consistently elevates p62 levels in multiple cell models, including 661W photoreceptor cells. Accordingly, the observed increase in cytoplasmic p62 immunoreactivity in 661W cells suggests lysosomal dysfunction and confirms autophagic inhibition, likely associated with the build-up of undegraded proteins (Yamamoto et al., 1998; Mizushima et al., 2011; Balmer et al., 2013; Klionsky et al., 2016). Our findings suggest that impaired proteostatic mechanisms may influence intracellular handling of Cx36 under stress conditions; however, direct conclusions regarding Cx36 turnover require additional functional assays. Our findings primarily reflect changes in proteostatic pathway markers rather than direct modulation of Cx36 turnover or localization.

In addition to elevated p62 expression, we also observed a marked upregulation of PSMA1-an α-subunit of the 20S proteasome core-in 661W photoreceptor cells following bafilomycin A1 treatment.

This finding is consistent with reports suggesting that pharmacological inhibition of lysosomal degradation may be associated with compensatory potential involvement of the UPS. Specifically, prolonged autophagic-lysosomal blockade by bafilomycin A1 has been shown to impair autophagic flux and simultaneously enhance expression of proteasome components such as PSMA1, reflecting an adaptive shift towards proteasomal degradation to manage accumulating misfolded proteins. Such proteasome upregulation likely represents a cellular response to preserve proteostasis when lysosomal clearance is compromised. While this suggests a potential compensatory involvement of the proteasome, immunofluorescence alone cannot confirm functional potential involvement - without functional proteasome or trafficking assays, these interpretations remain speculative (Tian et al., 2014; Oroń et al., 2022).

We observed an increase in RAB11A expression in 661W cells, suggesting activation of the recycling endosome pathway. RAB11A, a small GTPase localized to recycling endosomes, plays a central role in membrane transport and endosome-lysosome homeostasis. Impairment of lysosomal degradation-such as that induced by bafilomycin A1-leads to the intracellular accumulation of undegraded cargo, which may in turn stimulate RAB11A-mediated recycling process. This observation may reflect an adaptive cellular response associated with altered intracellular trafficking of proteins, including Cx36. Consequently, elevated RAB11A could reflect a compensatory mechanism in response to autophagy inhibition, potentially contributing to altered intracellular trafficking and protein redistribution under stress conditions. However, these phenomena warrant further investigation using trafficking assays or live-cell imaging to validate and extend our observations (Hu et al., 2015; Zulkefli et al., 2019).

In the retinal pigment epithelium (RPE), high metabolic activity and continuous phagocytosis expose cells to oxidative stress (OS), which disrupts proteostasis and leads to damaged organelle accumulation. Autophagy and endocytic pathways are essential to clear these aggregates; however, with aging or in conditions like age-related macular degeneration (AMD), lysosomal degradation becomes impaired, causing lipofuscin buildup and ROS elevation that exacerbate RPE dysfunction.

Moreover, autophagy interfaces with the UPS to maintain protein quality control in retinal neurons under oxidative challenge. OS-induced protein misfolding activates chaperone-mediated autophagy and the UPS; yet chronic OS hampers proteasomal activity, leading to aggregate accumulation and neuronal damage.

In retinal ganglion cells, moderate oxidative stress induces autophagy as a neuroprotective response, facilitating mitophagy and reducing ROS levels. However, when autophagic flux is exhausted-or proteasomal degradation is compromised-misfolded protein accumulation triggers neurodegeneration via pathways such as axonal degeneration and apoptotic signaling (Zhang Z. Y. et al., 2020; Chang et al., 2022; Markitantova and Simirskii, 2025; Wu et al., 2025).

Retinal diseases such as AMD and glaucoma are characterized not only by disruptions in retinal signaling and photoreceptor integrity but also by progressive dysfunction and death of RGCs. In AMD, impaired signal transmission across photoreceptors and retinal pigment epithelium contributes to vision loss due to degenerative changes in the outer retina. In glaucoma, elevated intraocular pressure and neuroinflammation trigger axonal transport disruption, mitochondrial dysfunction, and calcium dysregulation-culminating in RGC apoptosis. Chronic glial potential involvement further exacerbates these processes by releasing neurotoxic mediators that compromise the inner retinal environment. Together, these pathological events underpin the progressive loss of retinal function in both AMD and glaucoma (Almasieh et al., 2012; Yang and Sun, 2023; Shen et al., 2023; Tsung et al., 2023; Cui et al., 2025). Our work contributes to the understanding of the mechanisms underlying retinal diseases as well as disturbances in signaling and impulse conduction within the visual system. Better understanding of subcellular mechanisms involved will potentially allow in the future to design precisely targeted pharmacological treatment. Although our findings may have implications for diseases such as glaucoma and AMD, these connections remain speculative and require validation in retinal explants or *in vivo* studies. Overall, our findings provide preliminary insight into the proteostatic regulation of Cx36 under stress conditions. However, due to the use of an *in vitro* model and primarily immunofluorescence-based analysis, these observations should be interpreted with caution. Further studies, including functional assays and *in vivo* validation, are required to confirm the proposed mechanisms and their relevance to retinal neurodegenerative diseases. Additionally, the use of Cx36 overexpression may itself influence intracellular proteostasis and should be considered when interpreting the observed changes in ubiquitin accumulation and degradation pathways.

A key strength of this study is the use of a photoreceptor-derived cell model to investigate the relationship between connexin expression and intracellular proteostatic pathways under controlled stress conditions. The combination of lysosomal inhibition and excitotoxic stimulation provides a complementary approach to assess distinct mechanisms regulating Cx36 turnover. Additionally, the combined use of lysosomal inhibition and excitotoxic stimulation allowed us to explore complementary aspects of proteostatic stress, providing a broader perspective on intracellular pathways regulating Cx36 handling.

It is important to emphasize that the present study focused on the subcellular localization of connexin 36 and related proteostatic markers, as revealed by immunofluorescence staining. While this method provides unique spatial resolution for assessing intracellular distribution, it does not allow for precise quantification of total protein expression. Future work will incorporate complementary biochemical approaches, including Western blotting and LC3-II flux assays and proteasome activity measurements. To validate and extend the present findings.

## Data Availability

The original contributions presented in the study are included in the article/supplementary material, further inquiries can be directed to the corresponding author.
