# Supplementary material for: Gap junction protein connexin 36 proteostatic mechanisms in photoreceptor cells subjected to excitotoxic stress
Source: Acta Biochim Pol. 2026 Aug 14;73:16091. doi: 10.3389/abp.2026.16091 (PMC13523667; doi:10.3389/abp.2026.16091)
Supplement: Supplementary file 1 [file Supplementaryfile1.pdf]

Peer Review Report

Review Report on Gap Junction Protein Connexin 36 Proteostatic Mechanisms in Photoreceptor Cells Subjected to Excitotoxic Stress

Original Research, Acta Biochim. Pol.

Reviewer: Andrea Bighinati
Submitted on: 23 Jan 2026
Article DOI: 10.3389/abp.2026.16091

EVALUATION

Q 1 **Please summarize the main findings of the study.**

The manuscript from Katan M. et al. focuses on the gap junction protein Cx36 involvement in autophagy and misfolded protein response in the 661W cell line as a model of photoreceptor. Both autophagy and UPR are well characterized pathways and have relevant implications in retinal degenerations, like Retinitis Pigmentosa and Age-related Macular Degeneration. The authors found an impaired turnover of Cx36 after Bafilomycin treatment, which blocks autophagic flux, or NMDA-mediated excitotoxicity of transfected 661W cells. In this study, they used immunofluorescence analysis to quantify the principal markers of autophagy, p62/SQSTM1 in 661W cells treated with Bafilomycin and found decreased levels of p62 in cells transfected with Cx36-GFP, increased levels of proteasome activity. Moreover, the authors found increased levels of PSMA1, suggesting a possible compensation by the UPR response and reduced levels of RAB11A in transfected cells.

Q 2 **Please highlight the limitations and strengths.**

The work from Katan and colleagues shows an interesting role of Cx36 in the modulation of autophagy and UPR response in a cell model of photoreceptor. Since the accumulation of misfolded proteins in the photoreceptor plays a critical role during RP progression. While the work has some potential, it is not clear whether the aim is to clarify Cx36 turnover or its effect on autophagy and UPR. While data on 661W cells transfected with Cx36-GFP show an effect of Cx36 upregulation on autophagy inhibition mediated by Bafilomycin, they claim that impaired proteostatic mechanisms under excitotoxic stress may alter intracellular turnover of Cx36. In the results, they did not observe any change in Cx36 turnover with NMDA mediated excitotoxicity. The investigation on subcellular localization of Cx36 in 661W cells was never mentioned in their results, and the ubiquitin staining performed showed an increase of ubiquitin only when the Cx36-GFP plasmid was transfected in the 661W cells. These results are very confusing since in the discussion, the authors mention that Cx36 levels are increased during excitotoxic stress and that knock-out of Cx36 provides protection from neuronal degeneration.

Q 3 **Please comment on the methods, results and data interpretation. If there are any objective errors, or if the conclusions are not supported, you should detail your concerns.**

The methods are not clearly detailed and lack essential information for experimental replication (e.g. plasmid used for transfection, precise description of treatment time, respective controls, and resolution used for imaging). The immunofluorescence for Cx36 is not convincing, since this connexin should mainly be located on the membrane of the cells, while from the images seem to be more cytoplasmic or nuclear. The statistical analysis is poorly described with no post-hoc test, but from the results, the comparison was made between groups for each treatment. The analysis of immunofluorescence intensity is not described. Was a background subtraction performed? Were ROIs used, or was the intensity from the entire image? Why are the results not normalized on the total cell number? Why did the authors present results on ESI and the method to treat 661W?

In my opinion, the conclusion regarding Cx36 turnover or its subcellular localization is not supported by the data obtained. No colocalization analysis with ubiquitin was performed, nor compartmental analysis with subcellular markers (e.g. EEA1). Moreover, Cx36, as a connexin, should be mainly located on the cell membrane, but the staining for this protein was mainly cytoplasmic with or without transfection. In addition, treatments with either Bafilomycin or NMDA were performed 24 hours after transfection and collected respectively after 12 and 4 hours. While controls (untreated or not transfected) were collected 48 hours after transfection. Treatment timing should be the same for experimental and control conditions. Lastly, transfection itself alters the ER clearance, overloading this compartment. To test their hypothesis, the authors should use an expression plasmid without Cx36 as a control.

Check List

Q 4 **Please provide your detailed review report to the editor and authors (including any comments on the Q4 Check List)**

The manuscript from Katan M. et al. focuses on the gap junction protein Cx36 involvement in autophagy and misfolded protein response in the 661W cell line as a model of photoreceptor. Both autophagy and UPR are well characterized pathways and have relevant implications in retinal degenerations, like Retinitis Pigmentosa and Age-related Macular Degeneration. Whereas the scope of the manuscript is clear and well explained, I have major concerns regarding their conclusions. The results produced in this work do not support the modification of Cx36 turnover in 661W cells, either by blocking autophagic flux or by inducing excitotoxic stress. The results under autophagy blocking are only related to autophagy markers and do not investigate Cx36 levels or distribution in the cells. This analysis was performed only with NMDA treatment, but the authors did not observe any difference in Cx36 positivity with or without NMDA. The modification in subcellular localization is also not supported by the authors' data, since a transfection with a strong promoter is performed on the 661W cells, the cellular localization of Cx36 is mainly in the ER compartment and cytoplasm, while it should be on the cell membrane. Moreover, no colocalization analyses were performed on Cx36 and the autophagic or UPR markers.

Lastly, it is not clear whether Cx36 promotes UPR response or autophagy, since from p62 quantification, it seems that increasing Cx36 levels by transfection, the authors observe a reduction of p62 and RAB11A under Bafilomycin, accordingly with reduced autophagy activation. On the other hand, they also claim (but data are not shown) that Cx36 increased levels increase PSMA1, supporting increased proteasome response. In addition, data on ubiquitin quantification are not convincing. The staining for ubiquitin in transfected cells is not shown, and the interpretation of the results could be misleading for the conclusions, since the effect on ubiquitin level could be related to transfection itself. I suggest adding a control group for transfection without Cx36. In the end, the authors did not investigate Cx36 localization or turnover in 661W cells, but observed only the modification in effectors of autophagic/UPR response after noxious stimuli that themself cause modifications in the effector selected.

Minor concerns:

- Overall, the manuscript is very disorganized, the images are not in a proper order, and the conclusions do not follow the results obtained by the authors.
- The introduction is very long and repetitive; the aim of the work is reported in different parts of the introduction.
- The ESI effect on Cx36 was not investigated in this work. I suggest not mentioning it at all.
- In line 77: citations are not merged
- Line 86: By connecting retinal neurons of different functions; replace with By connecting retinal neurons with different functions.
- In line 99: citations are not merged
- Line 108: I don't understand this statement. There are several immortalized cell lines for neurons. The 661W is one of them, and they report it in the next sentence
- In line 126: citations are not merged
- In line 137: citations are not merged
- In line 143: wrong position for a citation, since the phrase is their claim
- Lines 144-158: no citations for the sentences
- Line 163-164: "ATP facilitates the activation of ubiquitin by the E1 ubiquitin-activating enzyme through covalent attachment." This sentence is not clear; both E1 and ubiquitin are phosphorylated to increase their activity.
- Line 171: citation needed
- Line 176: citation needed
- Line 215-216: Why mention that 661W cells were stained with anti-arrestin?
- Lines 222-232: I think this sentence should be removed
- Line 233: Clarify if cells were cultured on glass or plastic
- Line 234: serum-reduced should be removed
- Line 255: PFA concentration was 4 or 5%?
- All the antibodies used should have the catalogue number in the materials section
- Report the magnification used for imaging
- Describe in detail the imaging analysis (ROI, normalization on cell number), and the statistical analysis (post hoc test, data exclusion criteria) used in this study

- Line 291: 661W cells transfected
- Line 269 and Fig.3: the staining from transfected and untransfected cells looks similar. Can the authors add quantification by WB? The image in green is the same for Figure 8.
- Figures 4 and 5: Use letters for the different panels. Can the authors explain why the controls have only one panel while the treatment has 2 panels? What is the difference? Moreover, some cells do not express the Cx36-GFP construct and the expression seems different from the staining reported in Figure 3. Please clarify this. The image caption should be more descriptive, reporting the magnification scale bar resolution. In Figure 4, use PSMA1 instead of PSMA, which is a different protein.
- Figure 6: Use letters for each graph. Quantification of PSMA1 is missing. Some data do not have error bars. State the post hoc test used for ANOVA.
- Figure 7: The caption is not clear. Was the staining performed on transfected or untransfected cells? Report the magnification.
- Figure 8: This figure is never mentioned in the text. Was the staining for endogenous Cx36 and Cx36-GFP tag or for ubiquitin? It seems that endogenous Cx36 is nuclear after NMDA stress. Report the magnification.
- Figure 9: The expression of Cx36 seems as strong as in untransfected cells. Report magnification.
- Line 376: I suggest removing this paragraph.
- Lines 389-391: Then you should observe an increased ubiquitin signal also in untransfected cells, since the expression of Cx36 is not changed, but in your experimental setup, you treat 661W with NMDA to mimic excitotoxic stress.
- Lines 415-417: I don't understand. The authors did not characterize the expression profile or the intracellular localization of Cx36 in this manuscript. Is there a reference for this claim?
- Line 418: "Under excitotoxic conditions, Cx36 expression is upregulated". From fig. 10, you demonstrate that NMDA treatment does not increase the expression of Cx36 in non-transfected cells.
- Line 451: Quantification of PSMA1 is missing.
- Line 503: no study on subcellular localization was performed.

Q 5 **Is the English language of sufficient quality?**

Yes.

Q 6 **Is the quality of the figures and tables satisfactory?**

No.

Q 7 **Does the reference list cover the relevant literature adequately and in an unbiased manner?**

Yes.

Q 8 **Are the statistical methods valid and correctly applied? (e.g. sample size, choice of test)**

No.

Q 9 **Are the methods sufficiently documented to allow replication studies?**

No.

Q 10 **Are the data underlying the study available in either the article, supplement, or deposited in a repository? (Sequence/expression data, protein/molecule characterizations, annotations, and taxonomy data are required to be deposited in public repositories prior to publication)**

No.

Q 11 **Does the study adhere to ethical standards including ethics committee approval and consent procedure?**

Not Applicable.

Q 12 Have standard biosecurity and institutional safety procedures been adhered to?

Not Applicable.

QUALITY ASSESSMENT

Q 13 Originality

Q 14 Rigor

Q 15 Significance to the field

Q 16 Interest to general audience

Q 17 Quality of the writing

Q 18 Overall quality of the study
